# Expression of Immune Checkpoint-Associated Proteins for CD24, Siglec-10, CD47, and SIRPα in Breast Phyllodes Tumor

**DOI:** 10.3390/ijms27125556

**Published:** 2026-06-19

**Authors:** Eunah Shin, Ja Seung Koo

**Affiliations:** Department of Pathology, Yonsei University College of Medicine, Seoul 03722, Republic of Korea

**Keywords:** phyllodes tumor, breast neoplasms, immune checkpoint, CD47, SIRPα, Siglec-10, CD24, tumor microenvironment, prognosis, immunohistochemistry

## Abstract

This study aimed to investigate the expression of immune checkpoint-associated proteins in phyllodes tumors (PTs) and assess their clinicopathologic and prognostic significance. Surgical resection specimens from 200 patients were included, and the expressions of CD24, Siglec-10, CD47, and SIRPα in both the epithelial and stromal components of the tumor were assessed, with ≥1% positivity considered positive. Of 200 cases, 145 were benign, 44 borderline, and 11 malignant. The expressions of CD24, Siglec-10, CD47, and SIRPα in stromal cells increased with tumor grade: CD24 was associated with stromal cellularity, atypia, and mitosis; Siglec-10 with stromal cellularity and atypia; CD47 with stromal atypia and mitosis; and SIRPα with stromal overgrowth and atypia. While expressions of CD24, CD47, and SIRPα were associated with either shorter disease-free survival (DFS) or shorter overall survival, multivariate analysis identified stromal CD24 expression as an independent predictor of shorter DFS. Immune checkpoint-associated proteins, particularly stromal CD24, CD47, and SIRPα, are associated with adverse features and poor outcomes in PTs, implicating potential prognostic and therapeutic relevance.

## 1. Introduction

Phyllodes tumors, rare fibroepithelial neoplasms that account for <1% of all reported breast tumors [1], share significant histopathologic overlaps with fibroadenomas, belonging to the fibroepithelial tumor category, and frequently exhibit heterogeneous histologic features within the same lesion. This hinders accurate differential diagnosis [2]. To date, the literature remains inconsistent; however, the World Health Organization (WHO) classified phyllodes tumors into benign, borderline, or malignant [3]. Although most documented phyllodes tumors are benign, the rate of local recurrence ranges from 17% to 27% based on histologic grade; distant metastasis occurs in approximately 22% of all reported malignant cases [1,2,4,5]. Histologic grading of phyllodes tumors is determined by a combination of features, including stromal cellularity, stromal atypia, mitotic activity, stromal overgrowth, and tumor margins [2]. This grading system correlates with patient prognosis; however, it remains limited in accurately predicting individual clinical outcomes.

Advancements in tumor immune evasion techniques and understanding have emphasized the importance of “do not eat me” signaling pathways, via which tumor cells evade macrophage-mediated phagocytosis. Among these, the CD47–SIRPα and CD24–Siglec10 axes are key innate immune checkpoints [6,7,8,9]. CD47 and CD24 overexpression in tumor cells facilitates their interaction with SIRPα and Siglec10 on macrophages, respectively, thereby promoting the transmission of inhibitory signals responsible for suppressing phagocytic activity and facilitating immune evasion. These pathways are heavily involved in modulating tumor-associated macrophages (TAMs), major components of the tumor microenvironment that contribute to tumor growth, invasion, and metastasis. Importantly, blocking these signaling interactions restores macrophage-mediated clearance of tumor cells, demonstrating a significant therapeutic potential [6,7,8,9]. Nevertheless, the expression patterns and clinical implications of macrophage-related immune checkpoint proteins in phyllodes tumors remain largely unexplored. Therefore, to address this knowledge gap, this study aimed to investigate the expression of immune checkpoint-associated proteins, including CD24, Siglec-10, CD47, and SIRPα, in phyllodes tumors and to elucidate their clinical and biological implications.

## 2. Results

### 2.1. Basal Characteristics of Patients with Phyllodes Tumors

The baseline characteristics of the 200 patients with phyllodes tumors included in the analysis are summarized in Table 1: 145 were classified as benign, 44 as borderline, and 11 as malignant. Tumor size significantly increased with higher histologic grade (*p* = 0.001), and so did the rate of tumor recurrence with increasing grade (*p* < 0.001). All three cases with distant metastasis showed lung metastases.

### 2.2. Expression of Immune Checkpoint-Associated Proteins According to Phyllodes Tumor Grade

**Figure 1:**
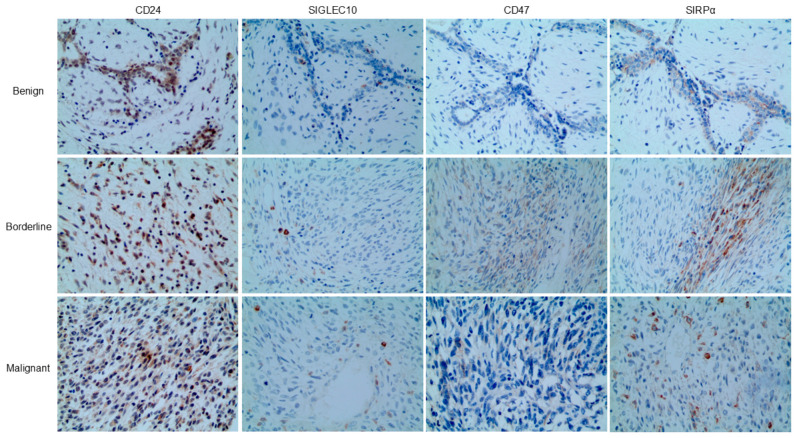
Expression of immune checkpoint-associated proteins in phyllodes tumors. Higher-grade phyllodes tumors (PTs) show increased expression of CD24, Siglec-10, CD47, and SIRPα in the stromal component.

Analysis of the expression of immune checkpoint-associated proteins by histologic grade of phyllodes tumors showed significant differences in stromal CD24 (*p* < 0.001), stromal Siglec-10 (*p* < 0.001), stromal CD47 (*p* = 0.008), and stromal SIRPα (*p* < 0.001). The proportion of positive cases for these markers significantly increased with higher tumor grade (Table 2 and Figure 1).

**Figure 2:**
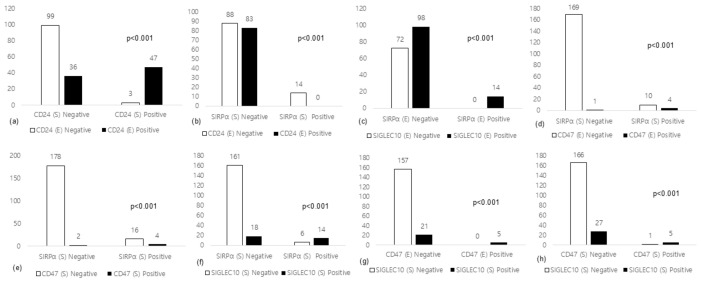
Associations among immune checkpoint-associated proteins. In the epithelial component, CD24 positivity is associated with a higher proportion of stromal CD24 positivity ((**a**), *p* < 0.001) and stromal SIRPα positivity ((**b**), *p* < 0.001). In the epithelial component, SIRPα positivity is associated with a higher proportion of epithelial Siglec-10 positivity ((**c**), *p* < 0.001). Stromal SIRPα positivity is associated with higher proportions of epithelial CD47 ((**d**), *p* < 0.001), stromal CD47 ((**e**), *p* < 0.001), and stromal Siglec-10 ((**f**), *p* < 0.001) positivity. In addition, stromal SIGLEC10 positivity correlates with higher proportions of epithelial CD47 ((**g**), *p* < 0.001) and stromal CD47 ((**h**), *p* < 0.001) positivity.

The results of the association analysis among immune checkpoint-associated proteins (Figure 2) showed that CD24 positivity in the epithelial component was significantly associated with higher positivity rates of CD24 and SIRPα in the stromal component (*p* < 0.001). SIRPα positivity in the epithelial component was significantly associated with a higher positivity rate of Siglec-10 in the epithelial component (*p* < 0.001). Furthermore, SIRPα positivity in the stromal component was associated with higher positivity rates of CD47 in both the epithelial (*p* < 0.001) and stromal components (*p* < 0.001), as well as Siglec-10 in the stromal component (*p* < 0.001). Siglec-10 positivity in the stromal component was significantly associated with increased positivity rates of CD47 in both the epithelial (*p* < 0.001) and stromal components (*p* < 0.001).

### 2.3. Correlation Between the Expressions of Immune Checkpoint-Associated Proteins and Clinicopathologic Parameters

**Figure 3:**
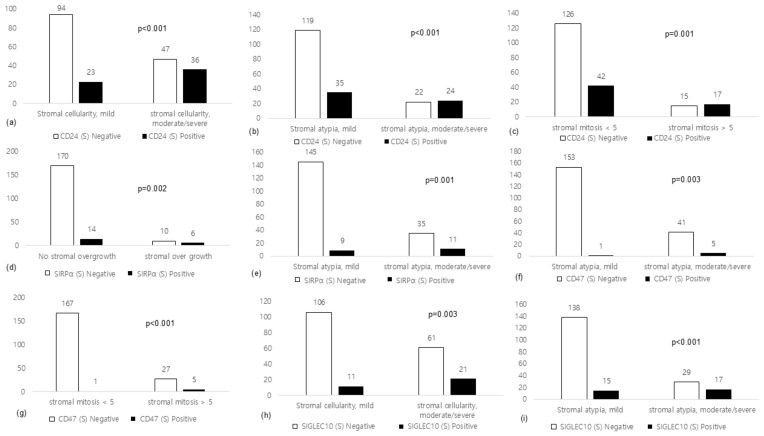
Correlation between immune checkpoint-associated protein expression and clinicopathologic parameters. Stromal CD24 status is significantly associated with stromal cellularity ((**a**), *p* < 0.001), stromal atypia ((**b**), *p* < 0.001), and stromal mitosis ((**c**), *p* = 0.001). Stromal CD24 positivity is associated with increased stromal cellularity, stromal atypia, and stromal mitosis. Stromal SIRPα positivity is associated with stromal overgrowth ((**d**), *p* = 0.002) and increased stromal atypia ((**e**), *p* = 0.001). Stromal CD47 positivity is associated with increased stromal atypia ((**f**), *p* = 0.003) and increased stromal mitotic activity ((**g**), *p* < 0.001). Stromal Siglec-10 positivity is associated with increased stromal cellularity ((**h**), *p* = 0.003) and increased stromal mitotic activity ((**i**), *p* < 0.001).

Stromal CD24 status was significantly associated with stromal cellularity (*p* < 0.001), stromal atypia (*p* < 0.001), and stromal mitosis (*p* = 0.001). Stromal CD24-positive phyllodes tumors were associated with high stromal cellularity, stromal atypia, and stromal mitosis. In addition, stromal SIRPα status was significantly associated with stromal overgrowth (*p* = 0.002) and stromal atypia (*p* = 0.001). Stromal SIRPα-positive phyllodes tumors were associated with elevated stromal atypia and stromal overgrowth. Stromal CD47 status was significantly associated with stromal atypia (*p* = 0.003) and stromal mitotic activity (*p* < 0.001), with stromal CD47-positive tumors showing increased stromal atypia and mitotic activity. Stromal Siglec-10 status was significantly associated with stromal cellularity (*p* = 0.003) and stromal atypia (*p* < 0.001). Stromal Siglec-10-positive tumors were associated with increased stromal cellularity and stromal mitotic activity (Figure 3).

### 2.4. Impact of Expression of Immune Checkpoint-Associated Proteins on Patients’ Prognosis

**Figure 4:**
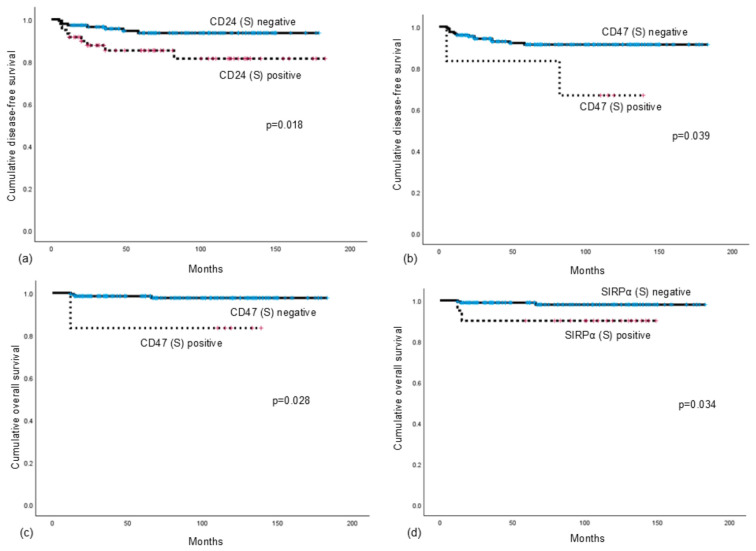
Impact of immune checkpoint-associated protein expression on patient prognosis. Univariate analysis demonstrates that stromal CD24 positivity is associated with shorter disease-free survival (DFS) ((**a**), *p* = 0.018). In addition, stromal CD47 positivity is associated with DFS ((**b**), *p* = 0.039) and shorter overall survival (OS) ((**c**), *p* = 0.028), while stromal SIRPα positivity is associated with shorter OS ((**d**), *p* = 0.034).

Univariate analysis was performed to evaluate the prognostic impact of immune checkpoint-associated protein expression in patients with phyllodes tumors. Stromal CD24 status was significantly associated with disease-free survival (DFS; *p* = 0.018), showing a correlation with shorter DFS. Stromal CD47 status was significantly associated with both DFS (*p* = 0.039) and overall survival (OS; *p* = 0.028), while stromal SIRPα status was associated with OS (*p* = 0.034). Stromal CD47 positivity was correlated with shorter DFS and OS, whereas stromal SIRPα positivity was associated with shorter OS (Table 3 and Figure 4).

In multivariate Cox proportional hazards analysis, independent factors associated with shorter DFS included stromal CD24 positivity (HR: 2.717, 95% CI: 1.028–7.183, *p* = 0.044, Table 4).

## 3. Discussion

In this study, we comprehensively evaluated the expression of the macrophage-associated innate immune checkpoint proteins CD24, Siglec-10, CD47, and SIRPα in phyllodes tumors (PTs) and demonstrated their presence in the epithelial and stromal components. Notably, the expression rates differed among the compartments: relatively higher Siglec-10 expression in the epithelial component and variable expression of CD47 and SIRPα across both compartments. Heterogeneous expression levels of CD24 and CD47 across tumor types have been previously reported and are likely attributable to differences in antibody clones and positivity thresholds. Importantly, Siglec-10 and SIRPα are predominantly expressed in tumor-associated macrophages (TAMs) within the tumor microenvironment (TME), rather than in tumor cells. Therefore, our observation of their expression in stromal components raises important biological questions regarding the cellular source of this expression.

PTs are biphasic fibroepithelial neoplasms in which the stromal component influences tumor behavior. Consistent with this concept, our results demonstrate a significant association between stromal expression of immune checkpoint-associated proteins, rather than epithelial expression, and higher histologic grade, adverse pathologic features (such as stromal cellularity, atypia, and mitotic activity), and poor clinical outcomes. These findings are consistent with those of previous studies emphasizing the central role of stromal biology and TAMs in PT progression [10,11,12]. Increased macrophage infiltration and stromal proliferation are linked to aggressive tumor behavior, supporting the biological plausibility of our observations.

An important finding of this study is that stromal tumor cells might exhibit macrophage-like features. The expression of macrophage markers, such as CD68 and CD163, in PT stromal cells suggests a degree of phenotypic plasticity [13]. In this context, the expression of Siglec-10 and SIRPα in stromal components might not be limited to infiltrating immune cells but could also reflect intrinsic properties of stromal tumor cells. This suggests an active role for PT stromal cells in shaping an immunosuppressive microenvironment via mechanisms typically attributed to TAMs. Further studies with spatial and single-cell analyses remain warranted to clarify the precise cellular origin of these proteins.

The CD47–SIRPα axis is among the best-characterized innate immune checkpoints [6]. Overexpression of CD47, which functions as a “do not eat me” signal that inhibits macrophage-mediated phagocytosis through interaction with SIRPα, has been reported in various malignant tumors and is associated with poor prognosis, enhanced metastatic potential, and resistance to therapy [14,15,16,17]. Herein, our results showed a significant association between stromal CD47 expression and elevated stromal atypia, higher mitotic activity, and worse survival outcomes, and that stromal CD47 expression remained an independent predictor of shorter disease-free survival. Similarly, stromal SIRPα expression correlated with adverse histologic features and poorer overall survival. These findings suggest that activation of the CD47–SIRPα axis contributes to immune evasion and tumor progression in PTs, paralleling its established role in other malignancies.

The CD24–Siglec-10 axis represents another emerging innate immune checkpoint pathway [9]. CD24 interacts with Siglec-10 on macrophages to suppress phagocytosis, functioning as an additional “do not eat me” signal. In this study, stromal Siglec-10 expression increased with tumor grade and was associated with key histologic parameters of aggressiveness. Furthermore, we observed significant correlations among CD47, SIRPα, and Siglec-10 expression, suggesting coordinated activation of multiple immune evasion pathways. Similar co-expression patterns have been described in other cancers, in which tumors employ redundant mechanisms to evade immune surveillance. These findings imply that high-grade PTs rely on multiple, overlapping innate immune checkpoint pathways, potentially contributing to resistance to single-agent immunotherapeutic strategies.

Clinically, these results have important implications. Current strategies targeting TAMs include inhibition of macrophage recruitment, macrophage depletion, reprogramming of macrophage polarization, and blocking the inhibitory signaling pathways [9]. Among these, targeting the CD47–SIRPα axis is a promising approach. Therapeutic modalities include monoclonal antibodies, small molecule inhibitors, gene therapy, and cellular therapies. Anti-CD47 antibodies such as magrolimab [18,19], evorpacept [20], and lemzoparlimab have demonstrated encouraging antitumor activity in both preclinical and clinical studies across various solid tumors. However, given the expression of CD47 on normal cells, such as erythrocytes, strategies, including bispecific antibodies, have been developed to improve tumor selectivity and reduce off-target toxicity [21].

Small molecule inhibitors targeting the CD47–SIRPα pathway offer an alternative approach, with the advantage of selectively inhibiting signaling without inducing broad immune activation [9].

These include agents that directly block CD47–SIRPα interaction (e.g., RS-17 and 4-oxadiazole compounds) as well as those that modulate pathway-related molecular processes (e.g., RRX-001 and PQ912), both of which have demonstrated promising antitumor properties [22,23,24,25]. Mechanistically, CD47–SIRPα axis inhibition promotes tumor suppression through multiple pathways, including enhanced macrophage-mediated phagocytosis, activation of dendritic cell-mediated antigen presentation, stimulation of T-cell responses, and augmentation of NK cell-mediated cytotoxicity [26,27,28].

Targeting the CD24–Siglec-10 axis offers another therapeutic avenue. Strategies include anti-CD24 monoclonal antibodies, gene-targeting approaches, and ligand–receptor binding disruption. In particular, antibodies such as SN3, SWA11, ML-5, ALB9, and G7mAb have been investigated, with SWA11 demonstrating antitumor efficacy in xenograft models by modulating tumor vasculature and cytokine environments and enhancing chemosensitivity [29,30]. This is particularly relevant for malignant PTs, which are traditionally resistant to chemotherapy and express cancer stem cell (CSC) markers [31,32,33]. In this context, combining anti-CD24-based therapies with conventional chemotherapy may represent a novel treatment strategy against malignant PTs.

This study has several limitations. Its retrospective design and single-institution cohort may limit generalizability; only 17 recurrence events and five deaths were observed in our cohort. Therefore, the multivariable Cox regression analyses, particularly those for overall survival, should be interpreted with caution because the limited number of events may increase the risk of model overfitting and instability of the estimated hazard ratios. Additionally, the use of tissue microarrays may not fully capture intratumoral heterogeneity. However, the key diagnostic feature of PTs is expansion and increased cellularity of the stromal component, rather than epithelial, and thus the intratumoral heterogeneity is relatively less than that of other types of tumors. Furthermore, functional validation of these immune checkpoint pathways was not performed, precluding definitive conclusions regarding causality. Future studies incorporating larger, multi-center cohorts, spatial profiling, and mechanistic experiments remain warranted to validate and extend these observations.

## 4. Materials and Methods

### 4.1. Patient Selection

This study included tissue samples from patients with PTs who underwent surgical resection at Severance Hospital between 2000 and 2013. Archived hematoxylin and eosin (H&E)-stained slides for each case were independently reviewed by two pathologists (J.S. Koo and E. Shin), and histologic grade was assessed according to the World Health Organization (WHO) classification [2]; accordingly, the tumors were grouped into benign, borderline, and malignant. Clinicopathologic data, including patient age, tumor recurrence, distant metastasis, and survival status, were collected and analyzed. The study was approved by the Institutional Review Board of Yonsei University Severance Hospital (IRB number: 4-2023-1370) on 11 December 2023.

### 4.2. Tissue Microarray

On the H&E-stained slide of the tumor, a representative area was selected, and the corresponding spot was marked on the surface of the paraffin block. Using a biopsy needle, the selected area was punched out, and the 3 mm tissue core was placed in a 5 × 6 recipient block. Two tissue cores were extracted to minimize extraction bias. Each tissue core was assigned a unique tissue microarray location number linked to a database containing other clinical-pathologic data. Although the use of two tissue cores may limit the capture of heterogeneity of the tumor components, the key diagnostic feature of PTs is expansion and increased cellularity of the stromal component, rather than epithelial, and thus the intratumoral heterogeneity is relatively less than that of other types of tumors, and two tissue cores are sufficient to represent the histologic features of the tumor.

### 4.3. Immunohistochemistry

The antibodies used for immunohistochemistry in this study are listed in Table 5. All immunohistochemical staining was performed on formalin-fixed, paraffin-embedded tissue sections using an automated staining system (Benchmark XT, Ventana Medical Systems, Tucson, AZ, USA). Briefly, 5 µm thick sections were mounted on adhesive slides and dried at 62 °C for 30 min. Heat-induced epitope retrieval was performed for 30 min in ethylenediaminetetraacetic acid (EDTA) buffer (pH 8.0) on the autostainer. The sections were incubated with primary antibodies. Following primary antibody incubation, the sections were treated with biotinylated anti-mouse immunoglobulins, then with peroxidase-labeled streptavidin using the LSAB kit (DakoCytomation, Glostrup, Denmark), and visualized with 3,3′-diaminobenzidine. Negative control samples were processed without primary antibodies, and appropriate positive control tissues were included according to the manufacturer’s instructions. All slides were counterstained with Harris hematoxylin.

Immunohistochemical staining was independently evaluated by two experienced pathologists blinded to the patients’ clinical information and outcomes. The expression of CD24, Siglec-10, CD47, and SIRPα was assessed in both the epithelial and stromal components of phyllodes tumors. A cutoff value of ≥1% positively stained cells was used to define positive expression for all markers. This threshold was selected based on its widespread use in immunohistochemical evaluation of clinically relevant biomarkers, including ER, PR, and PD-L1, where low-level expression may retain biological and clinical significance. In addition, the 1% cutoff provides greater sensitivity for detecting protein expression and has been employed in numerous exploratory immunohistochemical studies evaluating tumor-associated biomarkers.

### 4.4. Statistical Analysis

Data was analyzed using SPSS for Windows, Version 24.0 (SPSS Inc., Chicago, IL, USA). For the determination of statistical significance, Student’s *t*-test and Fisher’s exact tests were used for continuous and categorical variables, respectively. Statistical significance was defined as *p* < 0.05. Kaplan–Meier survival curves and log-rank tests were used to assess time to tumor recurrence. Multivariate regression analysis was performed using the Cox proportional hazards model.

## 5. Conclusions

The findings of this study demonstrate that macrophage-associated immune checkpoint proteins showed significantly different expression rates according to the histologic grade of the tumor. Particularly, CD47 and SIRPα were significantly associated with adverse stromal features, higher histologic grade, and poor clinical outcomes. The coordinated upregulation of CD47–SIRPα and CD24–Siglec-10 axes suggests that innate immune evasion plays a critical role in PT progression. These findings provide novel insights into the tumor biology of PTs and highlight potential prognostic biomarkers and therapeutic targets, supporting further investigation into macrophage-directed immunotherapies in this rare tumor type.

## Figures and Tables

**Table 1:** Clinicopathologic characteristics of patients with phyllodes tumors.

Parameters	Total N = 200 (%)	PT, Benign n = 145 (%)	PT, Borderline n = 44 (%)	PT, Malignant n = 11 (%)	*p*-Value
Age (years, mean ± SD)	40.2 ± 12.3	39.0 ± 12.2	42.8 ± 12.3	45.2 ± 9.9	0.079
Follow-up duration [months, mean (range)]	82.5 (12–183)	82.5 (14–183)	82.2 (12–144)	84.2 (12–135)	0.992
Tumor size (cm, mean ± SD)	3.9 ± 2.5	3.6 ± 2.2	4.3 ± 2.5	9.5 ± 5.0	**0.001**
Stromal cellularity					**<0.001**
Mild	117 (58.5)	113 (77.9)	3 (6.8)	1 (9.1)	
Moderate	70 (35.0)	32 (22.1)	38 (86.5)	0 (0.0)	
Marked	13 (6.5)	0 (0.0)	3 (6.8)	10 (90.9)	
Stromal atypia					**<0.001**
Mild	154 (77.0)	143 (98.6)	11 (25.0)	0 (0.0)	
Moderate	32 (16.0)	2 (1.4)	28 (63.6)	2 (18.2)	
Marked	14 (7.0)	0 (0.0)	5 (11.4)	9 (81.8)	
Stromal mitosis					**<0.001**
0–4/10 HPFs	168 (84.0)	145 (100.0)	23 (52.3)	0 (0.0)	
5–9/10 HPFs	20 (10.0)	0 (0.0)	19 (43.2)	1 (9.1)	
≥10/10 HPFs	12 (6.0)	0 (0.0)	2 (4.5)	10 (90.9)	
Stromal overgrowth					**<0.001**
Absent	184 (92.0)	145 (100.0)	38 (86.4)	1 (9.1)	
Present	16 (8.0)	0 (0.0)	6 (13.6)	10 (90.9)	
Tumor margin					**<0.001**
Circumscribed	176 (88.0)	142 (97.9)	33 (75.0)	1 (9.1)	
Infiltrative	24 (12.0)	3 (2.1)	11 (25.0)	10 (90.9)	
Tumor recurrence	17 (8.5)	5 (3.4)	9 (20.5)	3 (27.3)	**<0.001**

PT, Phyllodes Tumor; HPFs, high-power fields; Bold means *p* < 0.05.

**Table 2:** Expression of immune checkpoint-related proteins according to phyllodes tumor grade.

Parameters	Total N = 200 (%)	PT, Benign n = 145 (%)	PT, Borderline n = 44 (%)	PT, Malignant n = 11 (%)	*p*-Value
CD24 (E)					**0.003**
Negative	102 (55.1)	88 (61.5)	14 (35.9)	0 (0.0)	
Positive	83 (44.9)	55 (38.5)	25 (64.1)	3 (100)	
CD24 (S)					**<0.001**
Negative	141 (70.5)	114 (78.6)	24 (54.5)	3 (27.3)	
Positive	59 (29.5)	31 (21.4)	20 (45.5)	8 (72.7)	
Siglec-10 (E)					0.303
Negative	72 (39.1)	52 (36.4)	19 (50.0)	1 (33.3)	
Positive	112 (60.9)	91 (63.6)	19 (50.0)	2 (66.7)	
Siglec-10 (S)					**<0.001**
Negative	168 (84.0)	130 (89.7)	34 (77.3)	4 (36.4)	
Positive	32 (16.0)	15 (10.3)	10 (22.7)	7 (63.6)	
CD47 (E)					0.543
Negative	179 (97.3)	140 (97.9)	36 (94.7)	3 (100.0)	
Positive	5 (2.7)	3 (2.1)	2 (5.3)	0 (0.0)	
CD47 (S)					**0.008**
Negative	194 (97.0)	144 (99.3)	40 (90.9)	10 (90.9)	
Positive	6 (3.0)	1 (0.7)	4 (9.1)	1 (9.1)	
SIRPα (E)					0.721
Negative	171 (92.4)	132 (91.7)	36 (94.7)	3 (100.0)	
Positive	14 (7.6)	12 (8.3)	2 (5.3)	0 (0.0)	
SIRPα (S)					**<0.001**
Negative	180 (90.0)	136 (93.8)	38 (86.4)	6 (54.5)	
Positive	20 (10.0)	9 (6.2)	6 (13.6)	5 (45.5)	

PT, phyllodes tumor; E, epithelial component; S, stromal component; Bold means *p* < 0.05.

**Table 3:** Univariate analysis of the expression of PD-L1 with respect to patient prognosis using the log-rank test.

Parameters	No. of Patients Total/Recurrence/Death	Disease-Free Survival		Overall Survival	
		Median Survival (95% CI) Months	*p*-Value	Median Survival (95% CI) Months	*p*-Value
CD24 (E)			0.250		n/a
Negative	102/6/1	168 (161–176)		n/a	
Positive	83/8/0	164 (152–176)		n/a	
CD24 (S)			**0.018**		0.558
Negative	141/8/2	169 (162–175)		175 (171–179)	
Positive	59/9/3	154 (137–171)		177 (169–185)	
Siglec-10 (E)			0.857		n/a
Negative	72/5/0	163 (153–173)		n/a	
Positive	112/9/1	168 (159–177)		n/a	
Siglec-10 (S)			0.567		0.162
Negative	167/15/3	163 (155–170)		175 (172–179)	
Positive	32/2/2	172 (159–186)		172 (158–186)	
CD47 (E)			n/a		n/a
Negative	179/14/1	n/a		n/a	
Positive	5/0/0	n/a		n/a	
CD47 (S)			**0.039**		**0.028**
Negative	194/15/4	169 (162–175)		179 (175–182)	
Positive	6/2/1	107 (66–147)		117 (79–155)	
SIRPα (E)			n/a		n/a
Negative	171/14/1	n/a		n/a	
Positive	14/0/0	n/a		n/a	
SIRPα (S)			0.486		**0.034**
Negative	180/16/3	166 (159–174)		179 (176–183)	
Positive	20/1/2	141 (128–155)		135 (117–153)	

E, epithelial component; S, stromal component; n/a, not available; Bold means *p* < 0.05.

**Table 4:** Multivariate analysis of the disease-free survival and overall survival of patients with phyllodes tumors.

Included Factor	Disease-Free Survival			Overall Survival		
	Hazard Ratio	95% CI	*p*-Value	Hazard Ratio	95% CI	*p*-Value
CD24 (S)			**0.044**	Not included		
Negative vs. Positive	2.717	1.028–7.183				
CD47 (S)			0.154			0.321
Negative vs. Positive	3.004	0.668–13.609		3.545	0.291–43.106	
SIRPα (S)	Not included					0.186
Negative vs. Positive				3.968	0.515–30.590	

S, stromal component; Bold means *p* < 0.05.

**Table 5:** Source, clone, and dilution of the antibodies used.

Antibody	Company	Clone	Dilution
CD24	Abcam, Cambridge, UK	Polyclonal	1:200
SIGLEC10	Abcam, Cambridge, UK	EPR 29036-83	1:100
CD47	Abcam, Cambridge, UK	EPR 21794	1:100
SIRPα	Abcam, Cambridge, UK	EPR 22930-163	1:100

## Data Availability

All data pertaining to the study are comprehensively included in the article.

## Abbreviations

The following abbreviations are used in this manuscript:

PTs	phyllodes tumors
TAMs	tumor-associated macrophages
TME	tumor microenvironment
CSC	cancer stem cell
H&E	hematoxylin and eosin
EDTA	ethylenediaminetetraacetic acid
